# Supplementary material for: Spatial Transcriptomic Analysis Reveals Associations between Genes and Cellular Topology in Breast and Prostate Cancers
Source: Cancers (Basel). 2022 Oct 4;14(19):4856. doi: 10.3390/cancers14194856 (PMC9562681; doi:10.3390/cancers14194856)
Supplement: Supplementary file 1 [file cancers-14-04856-s001.zip › cancers-1924504-supplementary.pdf]

Supplementary Materials: Spatial Transcriptomic Analysis Reveals Associations between Genes and Cellular Topology in Breast and Prostate Cancers

Lujain Alsaleh, Chen Li, Justin L. Couetil, Ze Ye, Kun Huang, Jie Zhang, Chao Chen and Travis S. Johnson*

Supplementary Figures

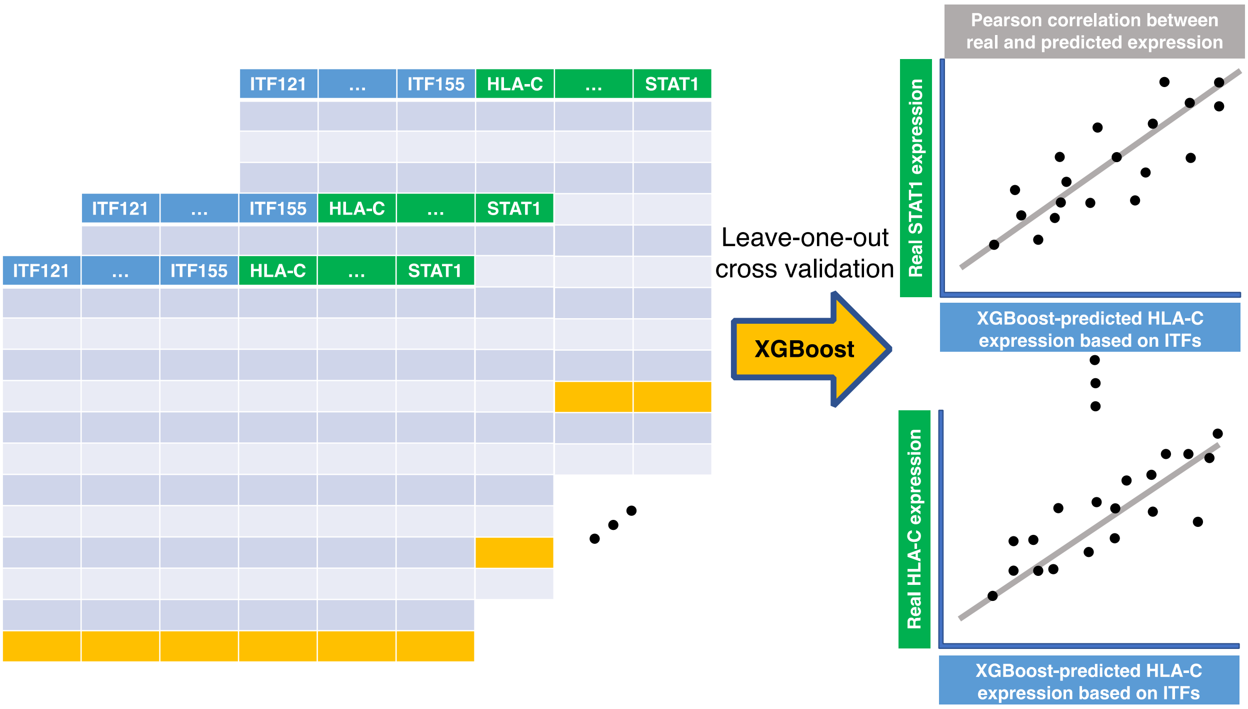

Figure S1. Pearson correlation between XGBoost-predicted gene expression based on image topology features and measured gene expression from ST.

Supplementary Tables

Table S1. Summary of correlation analysis and clustering results across all datasets and image feature types.

	1-dimensional Image Topological Features				0-dimensional Image Topological Features			
Slide ID	Minimum PCC	Maximum PCC	TAG clusters	ITF clusters	Minimum PCC	Maximum PCC	TAG clusters	ITF clusters
Parent Visium Human Breast Cancer	−0.05	0.25	6	7	−0.20	0.20	6	4
Parent Human Breast Cancer	−0.05	0.25	5	5	−0.05	0.25	6	5
FFPE Human Breast Cancer	−0.15	0.10	5	5	−0.15	0.10	5	5
Human Prostate Cancer	−0.40	0.40	5	4	−0.4	0.4	4	4
Prostate Acinar Cell Carcinoma	−0.15	0.15	5	5	−0.1	0.2	5	5
FFPE Human Normal Prostate	−0.2	0.4	5	4	−0.4	0.4	5	6

Table S2. Functional enrichment for TAGs in Parent Visium Human Breast Cancer Slide (1-dimensional Image Topological Features and 0-dimensional Image Topological Features) with the lowest significant *p*-values.

Term Id	Term Name	Adjusted *p*-Value	Gene Cluster Number
	**1-dimensional Image Topological Features**		
GO:0005615	extracellular space	$5.569958e^{-5}$	1
GO:0070062	extracellular exosome	$1.268685e^{-3}$	1
GO:1903561	extracellular vesicle	$1.409804e^{-3}$	1
GO:0043230	extracellular organelle	$1.409804e^{-3}$	1
GO:0065010	Extracellular membrane-bounded organelle	$1.409804e^{-3}$	1
HP:0001427	Mitochondrial inheritance	$5.697496e^{-8}$	2
HP:0002572	Episodic vomiting	$3.811384e^{-7}$	2
HP:0004309	Ventricular preexcitation	$9.907111e^{-7}$	2
HP:0003200	Ragged-red muscle fibers	$1.110454e^{-6}$	2
HP:0000576	Centrocecal scotoma	$1.664157e^{-6}$	2
GO:0002162	Dystroglycan binding	0.006281256	3
GO:0005584	Collagen type I trimer	0 .0003033615	4
HP:0005623	Absent ossification of calvaria	0.0126888764	4
HP:0003321	Biconcave flattened vertebrae	0.0126888764	4
HP:0005005	Femoral bowing present at birth, straightening with time	0.0126888764	4
HP:0005897	Severe generalized osteoporosis	0.0126888764	4
CORUM:6822	ZAG-PIP complex	0.04994746	5
	**0-dimensional Image Topological Features**		
GO:0005615	extracellular space	0.01631767	1
GO:0070062	extracellular exosome	$3.134382e^{-9}$	2
GO:1903561	extracellular vesicle	$3.774648e^{-9}$	2
GO:0065010	extracellular membrane-bounded organelle	$3.774648e^{-9}$	2
GO:0043230	extracellular organelle	$3.774648e^{-9}$	2
GO:0005615	extracellular space	$8.959959e^{-9}$	2
HP:0001427	Mitochondrial inheritance	$2.428127e^{-9}$	3
KEGG:05415	Diabetic cardiomyopathy	$1.142388e^{-8}$	3
HP:0004309	Ventricular preexcitation	$4.254401e^{-8}$	3
HP:0000576	Centrocecal scotoma	$1.239934e^{-7}$	3
HP:0002572	Episodic vomiting	$1.513199e^{-6}$	3
KEGG:05415	Diabetic cardiomyopathy	0.02883342	4
GO:0070069	cytochrome complex	0.03385070	4
KEGG:05012	Parkinson disease	0.03385070	4
KEGG:05010	Alzheimer disease	0.04709358	4
CORUM:6822	ZAG-PIP complex	0.04994746	5

Table S3. Functional enrichment for TAGs in Parent Human Breast Cancer Slide (1-dimensional Image Topological Features and 0-dimensional Image Topological Features) with the lowest significant *p*-values.

Term Id	Term Name	Adjusted *p*-Value	Gene Cluster Number
	**1-dimensional Image Topological Features**		
GO:0005615	extracellular space	$5.569958e^{-5}$	1
GO:0070062	extracellular exosome	$1.268685e^{-3}$	1
GO:1903561	extracellular vesicle	$1.409804e^{-3}$	1

GO:0043230	extracellular organelle	$1.409804e^{-3}$	1
GO:0065010	Extracellular membrane-bounded organelle	$1.409804e^{-3}$	1
KEGG:05415	Diabetic cardiomyopathy	$9.187864e^{-10}$	2
HP:0001427	Mitochondrial inheritance	$1.191038e^{-9}$	2
HP:0002572	Episodic vomiting	$2.432983e^{-8}$	2
HP:0000576	Centrocecal scotoma	$2.455963e^{-8}$	2
HP:0004309	Ventricular preexcitation	$3.506480e^{-8}$	2
GO:0002162	Dystroglycan binding	0.006281256	3
CORUM:6822	ZAG-PIP complex	00.04994746	4
	**0-dimensional Image Topological Features**		
GO:0005615	extracellular space	$5.569958e^{-5}$	1
GO:0070062	extracellular exosome	$1.268685e^{-3}$	1
GO:1903561	extracellular vesicle	$1.409804e^{-3}$	1
GO:0043230	extracellular organelle	$1.409804e^{-3}$	1
GO:0065010	Extracellular membrane-bounded organelle	$1.409804e^{-3}$	1
HP:0001427	Mitochondrial inheritance	$5.697496e^{-8}$	2
HP:0002572	Episodic vomiting	$3.811384e^{-7}$	2
HP:0004309	Ventricular preexcitation	$9.907111e^{-7}$	2
HP:0003200	Ragged-red muscle fibers	$1.110454e^{-6}$	2
HP:0000576	Centrocecal scotoma	$1.664157e^{-6}$	2
GO:0002162	Dystroglycan binding	0.006281256	3
GO:0005584	collagen type I trimer	0.0003033615	4
HP:0005623	Absent ossification of calvaria	0.0126888764	4
HP:0003321	Biconcave flattened vertebrae	0.0126888764	4
HP:0005005	Femoral bowing present at birth, straightening with time	0.0126888764	4
HP:0005897	Severe generalized osteoporosis	0.0126888764	4
CORUM:6822	ZAG-PIP complex	0.04994746	5

Table S4. Functional enrichment for TAGs in FFPE Human Breast Cancer Slide (1-dimensional Image Topological Features and 0-dimensional Image Topological Features) with the lowest significant *p*-values.

Term Id	Term Name	Adjusted *p*-Value	Gene Cluster Number
	**1-dimensional Image Topological Features**		
GO:0070062	extracellular exosome	$2.051973e^{-6}$	1
GO:1903561	extracellular vesicle	$2.362341e^{-6}$	1
GO:0065010	extracellular membrane-bounded organelle	$2.362341e^{-6}$	1
GO:0043230	extracellular organelle	$2.362341e^{-6}$	1
GO:0005615	extracellular space	$3.281412e^{-6}$	1
GO:0005615	extracellular space	$2.874349e^{-8}$	3
GO:0005576	extracellular region	$4.032521ee^{-6}$	3
GO:0070062	extracellular exosome	$7.746915e^{-6}$	3
GO:1903561	extracellular vesicle	$8.977896e^{-6}$	3
GO:0043230	extracellular organelle	$8.977896e^{-6}$	3
GO:0015453	oxidoreduction-driven active transmembrane transporter activity	$1.196315e^{-9}$	4
KEGG:05415	Diabetic cardiomyopathy	$3.163770e^{-9}$	4
GO:0098803	respiratory chain complex	$5.166699e^{-9}$	4

GO:0070469	respirasome	$1.073422e^{-8}$	4
HP:0002572	Episodic vomiting	$1.376587e^{-8}$	4
**0-dimensional Image Topological Features**			
GO:0005615	extracellular space	$2.175512e^{-5}$	1
GO:0070062	extracellular exosome	$7.189842e^{-5}$	1
GO:1903561	extracellular vesicle	$7.519775e^{-5}$	1
GO:0043230	extracellular organelle	$7.519775e^{-5}$	1
GO:0065010	extracellular membrane-bounded organelle	$7.519775e^{-5}$	1
HP:0000576	Centrocecal scotoma	$2.634312e^{-11}$	2
HP:0001427	Mitochondrial inheritance	$1.891416e^{-10}$	2
HP:0200125	Mitochondrial respiratory chain defects	$5.897102e^{-10}$	2
HP:0004309	Ventricular preexcitation	$2.038573e^{-9}$	2
HP:0007763	Retinal telangiectasia	$3.471255e^{-9}$	2
GO:0005615	extracellular space	$2.918838e^{-8}$	3
GO:0005576	extracellular region	$6.894013e^{-7}$	3
GO:0070062	extracellular exosome	$8.051882e^{-7}$	3
GO:1903561	extracellular vesicle	$9.590950e^{-7}$	3
GO:0065010	extracellular membrane-bounded organelle	$9.601088e^{-7}$	3
KEGG:05415	Diabetic cardiomyopathy	0.0000012396	4
GO:0005584	collagen type I trimer	0.0014950736	4
GO:0015453	oxidoreduction-driven active transmembrane transporter activity	0.0222768962	4
CORUM:2886	Respiratory chain complex I (incomplete intermediate ND1, ND2, ND3, CIA30 assembly), mitochondrial	0.0261210401	4
GO:0098803	respiratory chain complex	0.0429815125	4

Table S5. Functional enrichment for TAGs in Human Prostate Cancer Slide (1-dimensional Image Topological Features and0-dimensional Image Topological Features) with the lowest significant *p*-values.

Term Id	Term Name	Adjusted *p*-Value	Gene Cluster Number
**1-dimensional Image Topological Features**			
GO:0070062	extracellular exosome	$8.522469e^{-21}$	1
GO:1903561	extracellular vesicle	$1.427739e^{-20}$	1
GO:0065010	extracellular membrane-bounded organelle	$1.457071e^{-20}$	1
GO:0043230	extracellular organelle	$1.457071e^{-20}$	1
GO:0031982	vesicle	$1.842410e^{-20}$	1
MIRNA:hsa-miR-489-5p	hsa-miR-489-5p	0.01172711	2
GO:0010595	positive regulation of endothelial cell migration	0.03729422	2
REAC:R-HSA-445355	Smooth Muscle Contraction	$6.325223e^{-10}$	3
GO:0006936	muscle contraction	$1.717889ee^{-8}$	3
REAC:R-HSA-397014	Muscle contraction	$7.828103e^{-8}$	3
GO:0003012	muscle system process	$9.496920e^{-8}$	3
HPA:0490693	smooth muscle; smooth muscle cells[High]	$3.967884e^{-7}$	3
GO:0005584	collagen type I trimer	0.001492507	4
HP:0005623	Absent ossification of calvaria	0.003113332	4
HP:0005897	Severe generalized osteoporosis	0.003113332	4
HP:0003321	Biconcave flattened vertebrae	0.003113332	4

HP:0005005	Femoral bowing present at birth, straightening with time	0.003113332	4
TF:M07618_1	Factor: SRF; motif: CCTTWTATGGNN; match class: 1	0.007103503	5
TF:M01304_1	Factor: SRF; motif: NNCCAWAWAAGGV; match class: 1	0.007103503	5
TF:M12669_1	Factor: SRF; motif: TTNCCTTATWTGGNC; match class: 1	0.016334781	5
GO:0050998	nitric-oxide synthase binding	0.016997056	5
TF:M01007_1	Factor: SRF; motif: CNKNKCCTTATWTGG-NNNN; match class: 1	0.044725161	5
**0-dimensional Image Topological Features**			
GO:0070062	extracellular exosome	$6.479362e^{-22}$	1
GO:1903561	extracellular vesicle	$1.107932e^{-21}$	1
GO:0065010	extracellular membrane-bounded organelle	$1.132104e^{-21}$	1
GO:0043230	extracellular organelle	$1.132104e^{-21}$	1
GO:0031982	vesicle	$3.814630e^{-17}$	1
GO:0034599	cellular response to oxidative stress	0.01761702	2
GO:0062197	cellular response to chemical stress	0.01927010	2
GO:0006979	response to oxidative stress	0.02181185	2
REAC:R-HSA-445355	Smooth Muscle Contraction	$1.115693e^{-7}$	3
GO:0006936	muscle contraction	$6.454091e^{-7}$	3
REAC:R-HSA-397014	Muscle contraction	$2.791592e^{-6}$	3
GO:0003012	muscle system process	$2.840664e^{-6}$	3
HPA:0490693	smooth muscle; smooth muscle cells[High]	$4.850593e^{-6}$	3
GO:0005584	collagen type I trimer	0.001540888	4
HP:0003321	Biconcave flattened vertebrae	0.011601258	4
HP:0005005	Femoral bowing present at birth, straightening with time	0.011601258	4
HP:0005623	Absent ossification of calvaria	0.011601258	4
HP:0005897	Severe generalized osteoporosis	0.011601258	4

Table S6. Functional enrichment for TAGs in Prostate Acinar Cell Carcinoma Slide (1-dimensional Image Topological Features and 0-dimensional Image Topological Features) with the lowest significant *p*-values.

Term Id	Term Name	Adjusted *p*-Value	Gene Cluster Number
**1-dimensional Image Topological Features**			
GO:0070062	extracellular exosome	$1.093617e^{-17}$	1
GO:1903561	extracellular vesicle	$1.652514e^{-17}$	1
GO:0065010	extracellular membrane-bounded organelle	$1.678243e^{-17}$	1
GO:0043230	extracellular organelle	$1.678243e^{-17}$	1
GO:0031982	vesicle	$2.652779e^{-15}$	2
GO:0005615	extracellular space	0.0002065117	2
GO:0005576	extracellular region	0.0005853319	2
HPA:0471443	skin 2; fibrohistiocytic cells[High]	0.0134575912	2
GO:0070062	extracellular exosome	0.0139763283	2
GO:1903561	extracellular vesicle	0.0153151399	2
GO:0071751	secretory IgA immunoglobulin complex	0.0001962954	3
GO:0071749	polymeric IgA immunoglobulin complex	0.0001962954	3
GO:0071746	IgA immunoglobulin complex, circulating	0.0001962954	3
GO:0071745	IgA immunoglobulin complex	0.0001962954	3

GO:0070062	extracellular exosome	0.0004214256	3
REAC:R-HSA-445355	Smooth Muscle Contraction	0.001364539	4
HPA:0401322	rectum; enterocytes - Microvilli[≥Medium]	0.001378253	4
HPA:0401321	rectum; enterocytes - Microvilli[≥Low]	0.002072836	4
HP:0002579	Gastrointestinal dysmotility	0.002825241	4
HP:0030895	Abnormal gastrointestinal motility	0.002913245	4
GO:0005520	insulin-like growth factor binding	0.008985672	5
**0-dimensional Image Topological Features**			
GO:0044754	Autolysosome	0.0002318339	1
GO:0000323	lytic vacuole	0.0003481053	1
GO:0005764	Lysosome	0.0003481053	1
GO:0005773	Vacuole	0.0008109995	1
GO:0005767	secondary lysosome	0.0009158836	1
GO:0031982	Vesicle	$6.295632e^{-5}$	2
GO:0070062	extracellular exosome	$2.288019e^{-4}$	2
GO:1903561	extracellular vesicle	$2.636669e^{-4}$	2
GO:0043230	extracellular organelle	$2.636669e^{-4}$	2
GO:0065010	extracellular membrane-bounded organelle	$2.636669e^{-4}$	2
GO:0070062	extracellular exosome	$1.861833e^{-13}$	3
GO:1903561	extracellular vesicle	$2.639846e^{-13}$	3
GO:0065010	extracellular membrane-bounded organelle	$2.672183e^{-13}$	3
GO:0043230	extracellular organelle	$2.672183e^{-13}$	3
GO:0005615	extracellular space	$2.010480e^{-12}$	3
GO:0071745	IgA immunoglobulin complex	$4.459986e^{-6}$	4
GO:0071751	secretory IgA immunoglobulin complex	$4.459986e^{-6}$	4
GO:0071749	polymeric IgA immunoglobulin complex	$4.459986e^{-6}$	4
GO:0071746	IgA immunoglobulin complex, circulating	$4.459986e^{-6}$	4
GO:0005615	extracellular space	$1.141363e^{-3}$	4
REAC:R-HSA-445355	Smooth Muscle Contraction	0.001364539	5
HPA:0401322	rectum; enterocytes - Microvilli[≥Medium]	0.001378253	5
HPA:0401321	rectum; enterocytes - Microvilli[≥Low]	0.002072836	5
HP:0002579	Gastrointestinal dysmotility	0.002825241	5
HP:0030895	Abnormal gastrointestinal motility	0.002913245	5

Table S7. Functional enrichment for TAGs in Visium FFPE Human Normal Prostate Slide (1-dimensional Image Topological Features and 0-dimensional Image Topological Features) with the lowest significant *p*-values.

Term Id	Term Name	Adjusted *p*-Value	Gene Cluster Number
**1-dimensional Image Topological Features**			
GO:0070062	extracellular exosome	$1.603815e^{-13}$	1
GO:1903561	extracellular vesicle	$2.219204e^{-13}$	1
GO:0043230	extracellular organelle	$2.240562e^{-13}$	1
GO:0065010	extracellular membrane-bounded organelle	$2.240562e^{-13}$	1
GO:0005615	extracellular space	$8.853403e^{-10}$	1
GO:0062023	collagen-containing extracellular matrix	$6.700992e^{-5}$	2
GO:0031012	extracellular matrix	$3.520194e^{-4}$	2
GO:0030312	external encapsulating structure	$3.520194e^{-4}$	2
CORUM:2254	CTGF/Hcs24-actin(beta/gamma) complex	$2.231109e^{-3}$	2
GO:0005615	extracellular space	$5.403000e^{-3}$	2
GO:0043230	extracellular organelle	$9.806696e^{-8}$	3
GO:0005576	extracellular region	$3.037689e^{-6}$	3

GO:0070887	cellular response to chemical stimulus	$8.624522e^{-4}$	3
GO:0010033	response to organic substance	$8.624522e^{-4}$	3
GO:0071310	cellular response to organic substance	$8.624522e^{-4}$	3
REAC:R-HSA-445355	Smooth Muscle Contraction	$1.111645e^{-14}$	4
HPA:0490693	smooth muscle; smooth muscle cells[High]	$3.736946e^{-12}$	4
GO:0006936	muscle contraction	$8.389078e^{-12}$	4
REAC:R-HSA-397014	Muscle contraction	$3.763786e^{-11}$	4
GO:0015629	actin cytoskeleton	$4.049754e^{-11}$	4
**0-dimensional Image Topological Features**			
GO:0070062	extracellular exosome	$6.127363e^{-10}$	1
GO:1903561	extracellular vesicle	$7.797512e^{-10}$	1
GO:0065010	extracellular membrane-bounded organelle	$7.797512e^{-10}$	1
GO:0043230	extracellular organelle	$7.797512e^{-10}$	1
GO:0005615	extracellular space	$1.210568e^{-7}$	1
GO:0070062	extracellular exosome	$1.870788e^{-6}$	2
GO:1903561	extracellular vesicle	$2.204394e^{-6}$	2
GO:0065010	extracellular membrane-bounded organelle	$2.204394e^{-6}$	2
GO:0043230	extracellular organelle	$2.204394e^{-6}$	2
GO:0005615	extracellular space	$4.329608e^{-4}$	2
GO:0062023	collagen-containing extracellular matrix	0.001950355	3
GO:0031012	extracellular matrix	0.007442751	3
GO:0030312	external encapsulating structure	0.007442751	3
TF:M07618_1	Factor: SRF; motif: CCTTWTATGGNN; match class: 1	0.043463755	3
TF:M01304_1	Factor: SRF; motif: NNCCAWAWAAGGV; match class: 1	0.043463755	3
GO:0005615	extracellular space	$3.635334e^{-8}$	4
GO:0005576	extracellular region	$4.175812e^{-6}$	4
GO:0070062	extracellular exosome	$2.636948e^{-4}$	4
GO:1903561	extracellular vesicle	$2.993143e^{-4}$	4
GO:0065010	extracellular membrane-bounded organelle	$2.993143e^{-4}$	4
REAC:R-HSA-445355	Smooth Muscle Contraction	$1.111645e^{-14}$	5
HPA:0490693	smooth muscle; smooth muscle cells[High]	$3.736946e^{-12}$	5
GO:0006936	muscle contraction	$8.389078e^{-12}$	5
REAC:R-HSA-397014	Muscle contraction	$3.763786e^{-11}$	5
GO:0015629	actin cytoskeleton	$4.049754e^{-11}$	5

Table S8. Pearson correlation coefficients for predicted gene expression vs. true gene expression from XGBoost and LightGBM models.

Gene	LightGBM	XGBoost
HLA-C	0.5740936	0.5590423
HLA-E	0.5414152	0.5277748
CIRBP	0.5209353	0.5027015
STAT1	0.5194179	0.4905116
NFKBIA	0.5238297	0.5100563
DDX17	0.5535355	0.5467688
IFI44L	0.4880083	0.4724115
PFN1	0.5774745	0.5617051
GUK1	0.5128658	0.4948653
SERF2	0.5470236	0.5302537
SQSTM1	0.4924199	0.4821771

DDX5	0.5233507	0.5189854
HSP90AB1	0.5078897	0.4966268
HSPA8	0.5211847	0.5112679
TAGLN2	0.5142726	0.5042566
PRRC2B	0.4864269	0.4828521
NCL	0.5032294	0.4868199
IFITM3	0.5146741	0.5020230
UBB	0.5289144	0.5132720
